# Pre-implementation planning for a new personalised, dementia post-diagnostic support intervention: exploring the perspective of professional stakeholders

**DOI:** 10.1192/bjo.2024.733

**Published:** 2024-08-06

**Authors:** Ayesha Dar, Jessica Budgett, Sedigheh Zabihi, Ellenyd Whitfield, Iain Lang, Penny Rapaport, Bronte Heath, Margaret Ogden, Rosemary Phillips, Alexandra Burton, Laurie Butler, Danielle Wyman, Juanita Hoe, Jill Manthorpe, Sarah Morgan-Trimmer, Freya Koutsoubelis, Claudia Cooper

**Affiliations:** Division of Psychiatry, University College London, London, UK; Centre for Psychiatry and Mental Health, Wolfson Institute of Population Health, Queen Mary University London, London, UK; University of Exeter, Exeter, UK; Alzheimer's Society, London, UK; Anglia Ruskin University, Cambridge, UK; University of West London, London, UK; The Policy Institute at King's, King's College London, London, UK

**Keywords:** Dementia, integrated care, workforce, manualised interventions, prevention

## Abstract

**Background:**

Only a third of people with dementia receive a diagnosis and post-diagnostic support. An eight session, manualised, modular post-diagnostic support system (New Interventions for Independence in Dementia Study (NIDUS) – family), delivered remotely by non-clinical facilitators is the first scalable intervention to improve personalised goal attainment for people with dementia. It could significantly improve care quality.

**Aims:**

We aimed to explore system readiness for NIDUS–family, a scalable, personalised post-diagnostic support intervention.

**Method:**

We conducted semi-structured interviews with professionals from dementia care services; the Consolidated Framework for Implementation Research guided interviews and their thematic analysis.

**Results:**

From 2022 to 2023, we interviewed a purposive sample of 21 professionals from seven English National Health Service, health and social care services. We identified three themes: (1) potential value of a personalised intervention – interviewees perceived the capacity for choice and supporting person-centred care as relative advantages over existing resources; (2) compatibility and deliverability with existing systems – the NIDUS–family intervention model was perceived as compatible with service goals and clients’ needs, but current service infrastructures, financing and commissioning briefs constraining resources to those at greatest need were seen as barriers to providing universal, post-diagnostic care; (3) fit with current workforce skills – the intervention model aligned well with staff development plans; delivery by non-clinically qualified staff was considered an advantage over current care options.

**Conclusions:**

Translating evidence for scalable and effective post-diagnostic care into practice will support national policies to widen access to support and upskill support workers, but requires a greater focus on prevention in commissioning briefs and resource planning.

Around 944 000 UK people live with dementia. Only two-thirds are diagnosed,^1^ of whom less than half have received a care plan or care plan review.^2^ There are over 700 000 unpaid family carers for people living with dementia; many report high rates of emotional distress and morbidity.^3^ National policy prioritises support to maintain independence and well-being of people living with dementia and their carers. National Health Service (NHS) England's Well Pathway for Dementia and other initiatives stress the importance of supporting all people diagnosed to live as well as possible.^4^ The UK National Institute for Health and Care Excellence (NICE) dementia guidelines recommend offering ‘psychosocial and environmental interventions to reduce distress’, personalised strategies for behavioural and sleep disturbance and carer support.^5^ There is an implementation gap between national policy and the current reality that two-thirds of those living with dementia are either undiagnosed or receive no post-diagnostic support, primarily due to resource and workforce pressures.

A recent systematic review described evidence for interventions that improved functioning, or individualised outcomes (in which clients and carers select the most personally meaningful outcomes) in randomised controlled trials (RCTs) involving community-dwelling people with dementia. Successful interventions were delivered in-person by trained clinicians (occupational therapists, physiotherapists, psychologists).^6^ In a recent trial, an intensive programme of exercise and functional activity training did not improve activities of daily living, physical activity or quality of life in people with dementia, and the authors suggested considering alternative approaches to maintaining well-being.^7^ One promising approach is to tailor both treatment goals (outcomes) and therapy to personal priorities. Goal-oriented cognitive rehabilitation by nurses and occupational therapists improved self-rated goal attainment in people with mild to moderate dementia.^8^ Interventions that have increased quality time lived at home by people with dementia in RCTs have been personally tailored, and this need for adaptability might explain why only clinically trained professionals delivered them in trials.^9^ The US ‘MIND at Home’ intervention, which successfully reduced all-cause transition from home living when delivered by clinically trained staff in an RCT,^10^ is now being evaluated with involvement of non-clinically trained staff in delivery.^11^ As an alternative to existing evidence-based interventions, we co-designed the New Interventions for Independence in Dementia Study (NIDUS) – family), which can be facilitated by non-clinical staff, remotely or in person. It is designed to be cost-effective when delivered at scale. It is the first fully manualised intervention that is tailored to goals that dyads (people living with dementia and family carers) set, by selecting modules involving behavioural management, carer support, psychoeducation, communication and coping skills, enablement and environmental adaptations. NIDUS–family can be delivered to care partners with or without the person with dementia; dyads select which modules to cover, and the order (Fig. 1). The NIDUS–family theoretical model,^9^ logic model^12^ and pilot study^13^ are reported elsewhere. In an RCT, it improved attainment of family carer-rated personalised goals for people living with dementia in their own homes, relative to goal-setting alone.^14^ Pre-implementation research evaluates how well an evidence-based intervention might fit into contexts beyond the original trial, including changes that may be necessary to replicate trial findings, and potential constraints or barriers to full implementation. This learning can then inform planning for full implementation.^15^ We aimed to improve our understanding of challenges associated with implementing NIDUS–family, by deepening our awareness of ‘real world’ factors, through eliciting perspectives of those not involved in our original trial about how it might be implemented into practice.

**Fig. 1:**
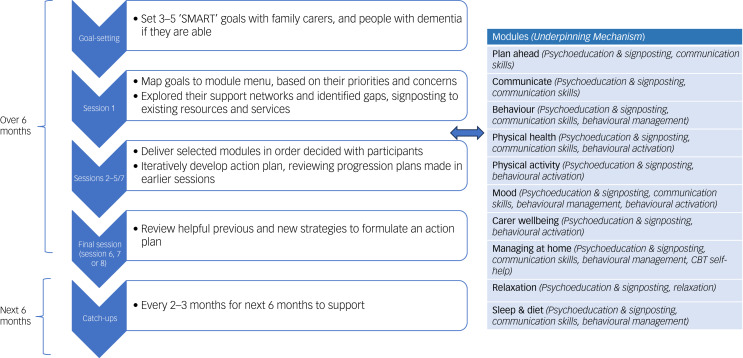
The New Interventions for Independence in Dementia Study (NIDUS) – family intervention structure. CBT, cognitive–behavioural therapy.

## Method

### Design and setting

We qualitatively interviewed participants from NHS primary and secondary care services, and third sector organisations commissioned to deliver health and care services throughout England, to explore how organisational and staff factors might influence their decisions around adopting NIDUS–family into practice. Sites were selected purposively to explore third sector and NHS, suburban, coastal and urban settings. Our NIDUS implementation group, comprising academic, public and patient involvement (PPI) representatives, policy and practice stakeholders, met throughout the NIDUS programme, to consider implementation during intervention development and testing. The group guided our sampling strategy and support interpretation of findings, situating the NIDUS programme in wider national policy and practice contexts.

### Ethics

The authors assert that all procedures contributing to this work comply with the ethical standards of the relevant national and institutional committees on human experimentation and with the Helsinki Declaration of 1975, as revised in 2008. All procedures involving human participants/patients were approved by the London-Camden and Kings Cross National Research Ethics Committees (19/LO/1667). The protocol (ISRCTN11425138) is published.^16^ Interview participants provided informed consent (written or audio recorded).

### Participant selection

Guided by the implementation group, we used a sampling framework purposively to select sites with different geographical and organisational contexts. Within sites, we sought to include diversity of roles and staff gender, if possible. We included senior staff with a leadership, managerial role in making decisions about dementia services, and frontline staff working with people living with dementia and/or their carers. Because, as explained above, we aimed to explore perspectives of those not engaged in the original trial, professionals from sites participating in the NIDUS–family trial were excluded. We identified potential participants through national networks of the implementation group, and support of the National Institute of Health Research (NIHR) clinical research network. Additional participants were identified through recommendations from interviewees (snowball sampling). We did not interview any implementation group members. Sample size was determined by our *a priori* intention to recruit a maximum variation sample, rather than concepts of data saturation.^17^

### Procedures

Interviews were conducted before NIDUS–family trial results^16^ were known. Interviewers asked participants to consider how NIDUS–family might be implemented, if it was demonstrated to be effective. In advance of the interviews, they were sent the participant information sheet and NIDUS–family intervention booklet (initial session, final session and two exemplar modules). At interview commencement, A.D. and J.B. summarised information about NIDUS–family rationale, programme and process. They were given the following explanation:

‘NIDUS–family aims to increase and sustain attainment of goals set by family carers and care recipients towards living as long and as well as possible at home. It is a fully manualised intervention that is tailored to these goals, by selecting modules developed in co-design workshops with PPI.^18^ Figure 1 illustrates the modules and delivery structure. NIDUS–family was delivered by video call/telephone (in person when Covid-19 restrictions permitted). Sessions included family carers and people living with dementia together, or just the family carer. The most appropriate arrangement was agreed with dyads (depending on their goals and circumstances) before each session. Facilitators in the main trial were not clinically trained but were trained and supervised by psychology or psychiatry clinicians’.^14^

Participants were then invited to ask questions about the study or NIDUS–family programme.

A.D. and J.B. used a semi-structured interview guide based on the Consolidated Framework for Implementation Research (CFIR). This theoretical framework can guide pre-implementation research, highlighting similarities and differences between and across settings.^19^ It includes five major domains – intervention characteristics, outer settings, inner settings, characteristics of the individuals involved and the process of implementation – encompassing 37 constructs.^19^ The topic guide (see Appendix 1) was created with the implementation group, and experts in implementation science and clinical care of people living with dementia. It focused on: the participant's role in their organisations and the services provided to people living with dementia; reflections on previous programmes delivered; staff, funding, support and training needed to implement NIDUS–family; and practicalities of implementing dementia care within the organisation. Questions varied by staff level (e.g. service manager questions focused more on staff and funding; frontline staff questions on support and training).

The interviewers (A.D. and J.B.) were NIDUS–family trial researchers. Interviews were conducted over Zoom or in person in a private area within the participants’ organisation. A.D. delivered NIDUS–family to participants in the trial and J.B. managed the trial. Interviews were audio recorded and transcribed verbatim, then de-identified prior to analysis. Interviews were 30–60 min in duration.

### Data analysis

We used an iterative approach to thematic analysis.^20^ We developed a codebook using the CFIR. Two researchers (A.D. and J.B.) deductively and inductively coded four of the same interviews and added new codes to the CFIR-based codebook best to reflect the content of the interviews.^21^ J.B. was the NIDUS programme manager and A.D. a researcher; both were women and in their 20s–30s. They thus held ‘insider perspectives’ of the NIDUS–family programme; analysis was planned for counterbalance. To do this, emerging findings were discussed with the NIDUS implementation group. A median of 13 members (range 8–21) attended eight meetings between March 2018 and September 2022; in addition to the research and NIDUS PPI representatives, members represented national (*n* = 3) and regional (*n* = 1) policymaking organisations, or were directors of local authorities (*n* = 2) or third sector care providers (*n* = 3) who were not directly involved in the NIDUS–family programme. Some of the group also read anonymised transcripts. Insider and outsider perspectives were acknowledged during analysis meetings, and in line with the purpose of the research, outsider perspectives were explicitly prioritised. Two researchers (A.D. and J.B.) then coded the remaining interviews using NVivo. The codes were together into similar topics with shared meaning to identify themes. Anonymised quotes were selected to illustrate themes, ensuring that quotes from all interviews were included.

## Results

### Qualitative interview participants

Data were collected between April 2022 and March 2023, in 20 interviews involving 21 participants. Two interviewees (9 and 12, who were colleagues) were interviewed together, at their request. We interviewed 11 managerial and ten frontline staff, from: (1) a national third sector provider of dementia specialist nurses (*n* = 1); (2) two local branches of a national social care provider in inner (*n* = 2) and outer (*n* = 2) London; (3) staff from the national office/helpline (*n* = 3) and local branches in South East (*n* = 1) and North West England (*n* = 2), outer London (*n* = 1) and West Midlands (*n* = −1) of a second national social care provider; (4) staff from a local social care provider (*n* = 3); (5) two NHS secondary care services (in urban (*n* = 1) and suburban (*n* = 3) areas); and (6) from primary care (*n* = 1). While we sought to recruit for diversity of staff background and roles, to minimise identifiability we did not collect information about participants’ age or ethnicity.

### Qualitative findings

We identified three main themes. The first, ‘Potential value as a personalised and adaptable intervention’, mapped to the Innovation domain of the CFIR and explores how interviewees considered NIDUS–family as having advantages over current care options. Our second theme, ‘Compatibility and deliverability with existing systems’, mapped to the inner setting domain; interviewees reported that NIDUS–family aligned with current service configurations, but resourcing and funding models would need to change before it could be universally offered as post-diagnostic care. Our third theme, ‘Fit with current workforce skills’, mapped to inner and outer setting domains, considering the advantages of NIDUS–family for staff and service development, and supervision and training needs to enable delivery by non-clinically trained staff. Table 1 describes interviewee characteristics (with interviewee numbers provided against quotes).

**Table 1:** Characteristics of interviewees

	No.	Role	Title	Gender
TSS 1 (commissioned for social care services)	1	Managerial	Area manager	Female
	2	Managerial	Head of service	Female
	3	Managerial	Area manager	Female
	4	Managerial	Head of service	Female
	5	Managerial	Local services manager	Male
	6	Front line	Dementia support worker	Female
	7	Front line	Dementia support worker	Male
	8	Front line	Dementia support worker	Male
TSS 2 (commissioned for social care services)	9	Managerial	Dementia safeguarding lead	Female
	10	Managerial	Head of prevention services	Female
	11	Managerial	Head of dementia care services	Female
	12	Managerial	Well-being lead	Female
NHS secondary care, suburban and rural	13	Front line	Senior support worker	Female
	14	Front line	Occupational therapy support worker	Female
	15	Managerial	Senior service line lead older adults	Male
NHS secondary care, urban	16	Front line	Consultant psychiatrist	Female
TSS (local) 3 (commissioned for social care services)	17	Front line	Senior support and well-being worker	Female
	18	Front line	Support and well-being worker	Female
	19	Managerial	Dementia action alliance coordinator	Male
NHS Primary care	20	Front line	Social prescriber	Female
TSS 4 (commissioned for health)	21	Front line	Specialist nurse	Female

No., number (of interviewee); TSS, third sector service; NHS, National Health Service.

#### Theme 1: potential value as a personalised and adaptable intervention

This theme mapped to CFIR intervention (innovation) constructs: source, design, adaptability and relative advantage. NIDUS–family was valued for its co-design by people with lived experience, providing flexible and accessible information and resources, delivery by a consistent and accessible facilitator, offering choice and providing individualised support. A need for linguistically and culturally adapted versions was identified.

##### Subtheme 1: innovation source

Several participants commented that NIDUS–family co-design by people with lived experience could increase confidence that it would be relevant to clients and carers. In the quote below, a dementia support worker suggests emphasising this when discussing the intervention with future adopters:

‘I would put … emphasis on the fact that this is being developed with people who have lived and experienced, because lots of people, if it doesn't expressly say that, they think, oh, it's just academics and professionals just sort of telling us what they think is right. But they don't know what it's really like in my life sort of thing. But you and I know that this is developed with people who are actually experiencing it, and if that can be sort of brought to the fore’ (third sector dementia support worker 7).

##### Subtheme 2: innovation design

All interviewees perceived NIDUS–family as potentially useful. In the following quote, an NHS support worker reflected, as did many interviewees, on how it presented information and resources in an accessible, manageable form:

‘I think it's quite invaluable because the way we work, quite often this information and support comes from lots of different organisations and voluntary organisations. But I think, to have something kind of all-in-one place almost like a learning resource for the family is quite invaluable, because I think it probably ties everything together and gives people an understanding and maybe the skills to manage their relative at home’ (senior NHS support worker 13).

Several frontline workers felt that family carers would value having an identified, accessible facilitator to talk to:

‘ … every service at the moment, [family carers are] calling and calling and trying to get through, but you've got someone coming down saying, I want to work with you. I can imagine it would be received, quite well’ (social prescriber 20).

‘ … it's the kind of thing that we would like to do because people often, not complain, but lament the fact that there is no continuity’ (dementia support worker 8).

##### Subtheme 3: innovation adaptability

Participants were enthused about adopting an intervention that included ‘choice’, ‘dialogue’ (third sector service lead) and person-centred care. An NHS service lead commented:

‘And I'm thinking sure, but we don't really do dialogue with people with dementia, but this feels very, very, person centred, a very Tom Kitwood [pioneer of person centred care] type way of being able to understand what's happening for a service user and then what can be done in order to engage them’ (NHS service lead 15).

Cultural adaptation of the intervention to languages other than English was suggested by several interviewees, to ensure the intervention fully encompassed local populations’ needs:

‘ … applicability to people from ethnic minority backgrounds in whom English may not be their first language and I think in London similar issues need to be considered just because we have a diverse population’ (NHS consultant psychiatrist 16).

‘we need to consider the needs of people whose first language is not English’ (third sector manager 3).

##### Subtheme 4: innovation relative advantage

Interviewees reflected on the potential utility of NIDUS–family to provide ‘the right tools to talk to people’ (third sector service lead 4). Relative advantages cited to current post-diagnostic care included provision of more and more individualised support, as demonstrated in a quote from one dementia support worker when comparing NIDUS–family with an existing intervention:

‘It's not as intensive as this, and it's not as individualised really, so I think something like this would be great’ (third sector dementia support worker 6).

The focus of the intervention on measuring change through specific, measurable, achievable, relevant, time-based (SMART) goals was an innovation welcomed by a specialist nurse:

‘Sometimes we feel that there's no change, and it just feels really tough. So, to actually be able to measure at baseline and after six sessions, some small change for the family and the clinician could be quite empowering, but also quite motivational’ (third sector specialist nurse 21).

#### Theme 2: compatibility and deliverability with existing systems

This theme captures how NIDUS–family was considered compatible with existing systems, maps to CFIR constructs in the inner (compatibility, relational connections, work infrastructure) and outer settings (external policy and incentives). Its delivery format aligned well with current systems, but current resources were perceived as insufficient to offer NIDUS–family as post-diagnostic care to all clients, with a need for policies and incentivisation to be adapted before such as person-centred post-diagnostic intervention could be provided as a universal offer.

##### Subtheme 1: compatibility

Health and social care professionals felt NIDUS–family facilitation by non-clinical support workers sat well with how they delivered post-diagnostic care:

‘We already have a post-diagnostic support service. That is provided by [provider name]. And it would be of that, it would be part of that kind of suite of post-diagnosis support’ (NHS senior service lead 15).

One participant explained that the option of video call delivery as a good fit to how their service had worked since the Covid-19 pandemic, and one that met family carers’ needs:

‘A lot of them don't have care, you know it's just they're on their own, and they don't have anyone to sit with the person they're caring for. So, for them Zoom is ideal, you know, because you know it's just like, it's low stress, you know’ (third sector service lead 19).

##### Subtheme 2: relational connections

A social prescriber perceived NIDUS–family as a potential tool towards their service goal of more integrated healthcare:

‘I feel like it would probably work alongside very similar to what we're doing … if the social prescribers and the, you know, the person facilitating were able to work quite well together … more streamlined, more effective, better outcomes, and yes, probably a lot less ping ponging of emails and admin between NHS, secondary care, primary care because we'd all be [on the same] page’ (NHS social prescriber, 20).

A service lead within a local authority commissioned social care service felt that her service was ‘well connected’ (third sector service lead 10) with the local primary care, other voluntary/third sector organisations and the NHS memory assessment service, and that, if within the commissioning brief, NIDUS–family would map well to their networks.

##### Subtheme 3: work infrastructure

Several interviewees reflected that resource constraints meant NIDUS–family could not be delivered to all clients as post-diagnostic support with current infrastructure. A psychiatrist, debating whether primary care might be able to accommodate it (and considering his secondary care service could not) commented:

‘I suppose you need the people to deliver the intervention. And I think that that is a stumbling block throughout NHS, where recruitment, and retention, are all such huge issues generally’ (NHS consultant psychiatrist 16).

An NHS support worker echoed the responses of many, that services were not currently resourced or commissioned to provide eight sessions of post-diagnostic support to everyone diagnosed, with their limited current resources targeted at those in most distress or need:

‘It's generally working with people that aren't settled and aren't stable and they've been referred to us for a period of intervention or work to try and for them to sort of settle say we're not sort of working with everybody with dementia that comes through the service, I am desperate to do that diagnostic support’ (NHS support worker, 14).

Interviewees were generally supportive of NIDUS–family, and considered how it might be adapted to align with current priorities. A support worker suggested targeting the intervention to those with greater needs:

‘I suppose if we're going to be spending that extra time with people it's about identifying the people that would really benefit and it might have to prevent somebody from them going into crisis and having that kind of intervention. So yes, I think if we could sort of identify people, and that would really sort of benefit from this who would require maybe a longer period of intervention. And then I think you know sort of offer five or six sessions incorporating maybe the contact that we would have with them as well, yeah that could be manageable’ (NHS support worker 14).

For one third sector manager, using the modules as a resource within care episodes as currently configured could help with the challenge of resourcing, albeit one that would create tension with delivering the core intervention as evaluated in the trial:

‘I think the way I would envisage it being used is that it would be another tool in the toolbox, and if the dementia support workers felt that actually a family would benefit from this intervention, then I think they would need to agree that because it would impact on their capacity to support other families’ (third sector manager 1).

##### Subtheme 4: external policy and incentives

While NHS interviewees discussed how the intervention might fit (or not) within current resources, social care interviewees perceived deliverability of NIDUS–family as contingent on whether it was commissioned:

‘I try and get money from commissioners, from CCGs (local NHS funders, clinical commissioning groups) and local authorities to implement our services. So, the more we have to sell you know, the better’ (third sector service lead 4).

‘So, we are working now locally with our commissioners, obviously, but also, now the commissioner landscape is changing, we've got meetings locally for us in [local city] with the new NHS integrated care system’ (third sector social care manager 5).

#### Theme 3: Fit with current workforce skills

Interviewees considered that NIDUS–family would fit well with current workforce skills and training, provided supervision was adequate (inner setting constructs). There were external drivers (outer setting) which could support its implementation (external pressures).

##### Subtheme 1: inner setting: access to knowledge and information

Managers and front-line staff described a learning climate that would align with adoption of NIDUS–family, perceiving it as a welcome opportunity for staff if included in their job roles:

‘Our dementia advisors are quite skilled … I think staff would buy it, you know. Of course, if they're doing NIDUS as part of case load, the case load would have to reduce. So, I think that, you know, if you were to speak to our DSWs [dementia support workers], I think they would love the opportunity to spend that much time with a client’ (third sector manager 1).

This was echoed in comments from support staff:

‘It would contribute to people's professional and personal development. If people had like a training booklet that they can keep referring back to, because we have so many different projects that each of us work on … I think it would really boost people's development’ (support worker 18).

‘We're very interested and passionate about improving somebody's quality of life. And so if it works, then I think most of us would be all for it. Also, just the – explaining it clearly enough to show myself and colleagues that it's not actually that complicated. You're not having to dedicate half your job to this, it's something that anyone can deliver’ (dementia support worker 8).

##### Subtheme 2: inner setting: available resources (supervision)

A specialist nurse highlighted the potential challenges for non-clinical staff of managing more complex cases, and the need for appropriate supervision and oversight:

‘I think in principle it's amazing, and it would work fantastically well with straightforward, non-high needs, non-complex families. And you may have to tweak it or add further safeguards to the package for those with greater needs … the intervention is encouraging that engagement. But as soon as that person's engaged, what else does that prompt as the relationship builds on that wouldn't be covered? And who would manage the extra needs that might need meeting?’ (third sector specialist nurse 21).

Front-line staff also emphasised the importance of clinical supervision and peer support sessions to discuss challenging situations:

‘I think some sort of supervision would definitely be beneficial. I think space to reflect on the different clients that were having an intervention. And I think if it was even just something very quick, weekly or fortnightly, depending on how often they were engaging, I think that would make a huge difference to the kind of success of the project’ (support and well-being worker 17).

Another potential supervision need was how to balance communication needs of the family carer and person living with dementia, when the intervention was delivered to dyads:

‘Juggling of two people not feeling heard, you know, the carer in their own sphere and perhaps thinking the person with dementia can't think about them and what they're going through and vice versa, um it can be very difficult terrain … to balance in providing support to both’ (third sector service lead 9).

##### Subtheme 3: outer setting: external pressures

At a time when allied health professionals are overstretched, policy and resource incentives to enhance the skills of non-clinical workers in support roles to deliver evidence-based interventions were attractive options to interviewees. A third sector support worker highlighted advantages in delivery by non-clinically trained facilitators, as a potential solution to lack of clinically trained professionals:

‘And also probably it would help people stay at home a lot longer because the carer is more educated, more aware of what they can do with things, especially when you look at the safety and adapting environments, you know, it's very hard to see occupational therapists here’ (support worker 7).

## Discussion

As NIDUS–family is, to our knowledge, the first evidence-based, modular, fully manualised post-diagnostic care intervention, it has potential to be more scalable than previous evidence-based programmes. This pre-implementation study provides useful insights into how it might move from research into practice. Interviewees perceived the intervention source and design positively, with relative advantages over existing interventions in the flexibility of delivery. They thought it aligned well with clients’ needs and appreciated its capacity to offer choice, support person-centred care, facilitate dialogue and focus care around goals. Interviewees identified a need for culturally and language adapted versions. Facilitators of implementation included potential compatibility with current systems (optimised for telephone and video call delivery, flexible and individualised approach), aligned learning environments, external pressures to develop evidence-based interventions and deliverability by a wider staff pool than qualified clinicians.

Barriers to implementation were within CIFR constructs of work infrastructure and external policy and incentives. Interviewees expressed concerns that current service configurations and resources lacked capacity to implement NIDUS–family as intended to everyone diagnosed with dementia and their families, although they perceived the potential clinical benefit and alignment with national policy. Social care professionals perceived NIDUS–family as deliverable if it fitted within future commissioning briefs.

There is a paucity of implementation research for community, non-pharmacological dementia care interventions, in a clinical area in which: ‘with few exceptions, proved interventions have not been translated for delivery in real-world settings’.^22^ It is a strength of the NIDUS–family trial that implementation was considered from the outset. Previous implementation and pre-implementation studies in this area focused on intervention, rather than organisational and wider contextual factors.^23,24^ One study explored implementation of a dementia case management intervention in Germany, identifying CFIR constructs of patients’ needs and resources, relative advantage and cosmopolitanism as potential barriers or facilitators.^25^ These described how the intervention lacked relevance for many clients, and that poor interfaces, especially digital interfaces between organisations were barriers to implementation. In a second study, reluctance of people with dementia to engage, for reasons including stigma, limited implementation of a sports project.^26^ A home arts programme described family carers as barriers or enablers to engagement, and hesitancy/suspicion among people living with dementia as barriers.^27^ The flexibility of NIDUS–family, including the option for sessions to be with the family carer alone, when the person living with dementia may be reluctant or unable to participate or lack insight, supported its implementation and was critical to its wide acceptability.

Previous community-based dementia implementation studies echo our findings regarding organisational factors: identifying staff training, external policy and incentives, time constraints and the need to integrate the intervention into existing systems as barriers.^23,24^ We used the CFIR-ERIC Matching tool to identify implementation strategies to circumvent these for NIDUS–family.^28,29^ The tool guided us to promote adaptability to maximise fit within existing systems (this might include identifying and preparing champions to explain adaptability to potential users) and to ‘access new funding’ and ‘consider other payment schemes’. Within the NHS and UK social care, this could include lobbying for inclusion of goal-focused post-diagnostic support within commissioning briefs, NICE guidelines and Memory Services National Accreditation Programme (MSNAP) quality improvement standards. We will make NIDUS–family manuals and training materials available online to facilitate its provision by the NHS and independent providers.

We hope that the good fit between NIDUS–family and England's current policy priorities of personalisation^30^ – integrating care,^31^ and aspirations of the NHS Long Term Workforce Plan^32^ to create new roles in the NHS workforce – will increase buy-in from national policymakers and commissioners. It could have particular relevance to the work of registered nursing associates, a new role bridging the gap between healthcare support workers and registered nurses,^32^ and support government social care reform plans by contributing to knowledge and skills frameworks and career pathways to support progression for social care workers.33 We are currently conducting an implementation study to translate NIDUS–family for the Bengali language and cultural context in east London, and to explore how NIDUS–family might work if delivered in social care and social prescribing contexts.

### Limitations

The clinical efficacy and cost-effectiveness of NIDUS–family were not available at the time of interviews. Subsequently, we have demonstrated clinical effectiveness;^14^ and await health economic evaluation. Our findings are based on research in the NHS and third sector settings in England and may not be generalisable to other health systems. Those agreeing to be interviewed are likely to have more positive attitudes towards early adoption of research findings, and there may have been a desirability bias in their responses. This pre-implementation analysis was by and not independent of the research team.

### Future directions

NIDUS–family aligns well with current service delivery models, national policy agendas,^30,31^ and was endorsed as a useful intervention with relative advantages over current care options. Most services are currently not resourced and commissioned to deliver person-centred post-diagnostic care of this intensity to everyone who could potentially benefit. Many interventions that improve the lives of research trial participants never move beyond research, potentially wasting scarce resources. If NIDUS–family is to deliver its full potential, changes to how care is funded and delivered are required. The introduction of NHS integrated care systems across England in 2022 was intended to change local commissioning, and the NIDUS–family model is a potentially useful tool to support implementation of more integrated care. Translating evidence for scalable and effective post-diagnostic care into practice will require a greater focus on prevention of morbidity and distress in commissioning briefs and resource planning. We will explore in future research how to support diffusion into practice of this effective intervention.

## Data Availability

The data that support the findings of this study are available from the corresponding author, C.C., on reasonable request.

## Appendix 1. Interview topic guide for frontline staff and senior staff

### NIDUS–family pre-implementation study topic guide for frontline staff

1. What do you think about interventions of this type, which are intended to help family carers of people with dementia?
2. Have you offered a one-to-one intervention like this within your organisation in the past? Yes – how does NIDUS seem to compare with similar interventions you have delivered in the past?
3. From what we've told you so far, is this something that you think you and your organisation could deliver as is, or would there need to be changes made before that could happen?
4. What adaptations need to be made to introduce this intervention? Probe for practicalities, geographical barriers, use of Zoom in rural set ups.
5. From your experience what are the key needs/gaps currently in this population, and what would be a priority for addressing these as well?
6. Who introduces new approaches in your organisation? Do you think your organisation would be open to introducing such an intervention?
7. What practical help or support might you need besides training?
8. In your experience, what factors contribute to high and low engagement with similar interventions from clients? Are there any issues that might make it easier or difficult?
9. What impact might an intervention like this have on your learning on a professional and a personal level?
10. What would encourage uptake of training among frontline members of staff?

### Topic guide for senior staff – pre-implementation study NIDUS–family

1. What do you think about interventions of this type, which are intended to help family carers of people with dementia?
2. Have you offered a one-to-one intervention delivered by non-clinical staff within your organisation in the past?
3. Yes – how was it introduced? No – is there anything that has got in the way of introducing similar interventions in the past?
4. What factors would need to be considered to help the intervention fit your organisation? Probe for time, money, availability of willing staff.
5. From what we've told you so far, is this something that you think you and your organisation could deliver as is, or would there need to be changes made before that could happen?
6. What adaptations to the service will your organisation need to make in order to introduce this intervention? Probe for practicalities, geographical barriers, use of Zoom in rural set ups.
7. Can you tell us about how changes to the service you provide have been made in the past? Follow-up to ask for an example, what made it harder or easier, etc.?
8. What practical help or support might your staff need besides training?
9. How would you ensure that the intervention is reaching the people that it's designed for, that is, people living with dementia/their caregivers?
10. How would you ensure that the people who are interested: (a) know about the intervention; and (b) join in with the intervention?
11. What are the key needs/gaps currently in the interventions being delivered by your organisation and what would be a priority for addressing these?
12. What qualities/outcomes of an intervention do you most value, and what outcomes make it suitable for routine use within your organisation? Are there any outcomes of similar interventions that you are asked to report on both internally within your organisation and to service commissioners?
13. What is it about these interventions that will make it more or less attractive?
14. Who would need to be involved in decision making/actions?
